# Knowledge translation: a case study on pneumonia research and clinical guidelines in a low- income country

**DOI:** 10.1186/1748-5908-9-82

**Published:** 2014-06-26

**Authors:** Sophie Goyet, Hubert Barennes, Therese Libourel, Johan van Griensven, Roger Frutos, Arnaud Tarantola

**Affiliations:** 1 Epidemiology and Public Health Unit, Institut Pasteur, Phnom Penh, Cambodia; 2 Agence Nationale de recherche sur le SIDA et les hépatites, Paris, France; 3 ISPED, Centre INSERM U897-Epidemiologie-Biostatistique, Université de Bordeaux, F-33000 Bordeaux, France; 4 INSERM, ISPED, Centre INSERM U897-Epidemiologie-Biostatistique, F-33000 Bordeaux, France; 5 Université Montpellier 2, UMR Espace Dev, IRD-UM2-UAG-ULR, Montpellier, France; 6 Sihanouk Hospital Center of HOPE, Phnom Penh, Cambodia; 7 Institute of Tropical Medicine, Antwerp, Belgium; 8 Université Montpellier 2, CPBS, UMR 5236 CNRS-UM1-UM2, Montpellier, France; 9 Intertryp, UMR 17, IRD-Cirad, Campus international de Baillarguet, 34398 Montpellier, Cedex 5, France

**Keywords:** Knowledge translation, Clinical practice guideline, Pneumonia, Low-income country

## Abstract

**Background:**

The process and effectiveness of knowledge translation (KT) interventions targeting policymakers are rarely reported. In Cambodia, a low-income country (LIC), an intervention aiming to provide evidence-based knowledge on pneumonia to health authorities was developed to help update pediatric and adult national clinical guidelines. Through a case study, we assessed the effectiveness of this KT intervention, with the goal of identifying the barriers to KT and suggest strategies to facilitate KT in similar settings.

**Methods:**

An extensive search for all relevant sources of data documenting the processes of updating adult and pediatric pneumonia guidelines was done. Documents included among others, reports, meeting minutes, and email correspondences. The study was conducted in successive phases: an appraisal of the content of both adult and pediatric pneumonia guidelines; an appraisal of the quality of guidelines by independent experts, using the AGREE-II instrument; a description and modeling of the KT process within the guidelines updating system, using the Unified Modeling Language (UML) tools 2.2; and the listing of the barriers and facilitators to KT we identified during the study.

**Results:**

The first appraisal showed that the integration of the KT key messages in pediatric and adult guidelines varied with a better efficiency in the pediatric guidelines. The overall AGREE-II quality assessments scored 37% and 44% for adult and pediatric guidelines, respectively. Scores were lowest for the domains of ‘rigor of development’ and ‘editorial independence.’ The UML analysis highlighted that time frames and constraints of the involved stakeholders greatly differed and that there were several missed opportunities to translate on evidence into the adult pneumonia guideline. Seventeen facilitating factors and 18 potential barriers to KT were identified. Main barriers were related to the absence of a clear mandate from the Ministry of Health for the researchers and to a lack of synchronization between knowledge production and policy-making.

**Conclusions:**

Study findings suggest that stakeholders, both researchers and policy makers planning to update clinical guidelines in LIC may need methodological support to overcome the expected barriers.

## Background

Pneumonia still imposes a major burden to populations and health systems in low-income countries (LIC) although interventions to prevent and treat pneumonia are known [1-4]. In Cambodia, a tropical LIC, pneumonia is the leading cause of death among children under five [5]. From 2007 to 2010, Institut Pasteur in Cambodia (IPC), a research agency, conducted a large study on pneumonia which included more than 4,000 patients of all ages [6,7]. This study describes the epidemiology of most acute lower respiratory infections treated in two major provincial hospitals [6-9]. Study findings were disseminated via various media targeting various audiences. However, in 2011, one year after the end of this study, those study findings were neither available to the Cambodian Ministry of Health (MoH) nor to the Cambodian physician in an easily accessible format. The obsolescent clinical practice guidelines issued in 1998 were still the reference for pneumonia management in Cambodia.

In August 2011, the MoH undertook the revision of the pediatric and adult guidelines for 180 diseases and health problems commonly treated in the country, including the management of community-acquired pneumonia [10,11]. This revision was given to two task forces of national clinicians coming from several MoH and nongovernmental organizations’ (NGO) hospitals.

Updating guidelines is a difficult task, particularly in LIC. Reliable guidelines must be epidemiologically relevant and reflect state-of-the art of medical practice. They have to be developed in a transparent way to facilitate their implementation and considering their acceptability and financial implications is also important [12]. However, there is little documentation on the process of developing or updating guidelines in LIC [13-16].

In September 2011, the IPC researchers were informed by the World Health Organization (WHO) Cambodian office that a revision of the national guidelines was being undertaken. Although there was no official demand for it from the MoH, the IPC researchers endeavored to provide the MoH with locally relevant and evidence-based knowledge on pneumonia. IPC researchers facilitated the creation of a multidisciplinary working group of national and international clinicians, biologists, health program managers and epidemiologists involved in pneumonia management in Cambodia, dubbed the CALIBAN network (‘Community-Acquired Lung Infections, Bacteria and Antimicrobial Network’) (List 1).

### List 1 Summary of the CALIBAN knowledge translation intervention

1. **Detailed description of the intervention**

a. **Characteristics of those who delivered the intervention**

• Intervention delivered by the CALIBAN group which included:

– European epidemiologists from a Cambodian-French research agency (n = 2)

– Cambodian clinicians from the Ministry of Health (n = 4)

– Cambodian clinicians and biologists from the National Institute of Public Health (n = 1)

– Cambodian and international clinicians and biologists from NGO hospitals (n = 8)

– International clinician affiliated with the University of Health Sciences (n = 1)

– European clinicians from a Cambodia-based research agencies (n = 1)

– Foreign health program managers from the World Health Organization (n = 1)

• Among the Cambodian clinicians, were the two experts in charge of drafting the updated appointed to review the pediatric and the adult pneumonia guidelines.

– One of them, a pulmonologist, agreed to chair the CALIBAN group.

– Another CALIBAN Cambodian clinician was also a member of the Task force in charge of updating the guidelines.

b. **Characteristics of the intervention’s targets**

• National Health program managers, policymakers and clinicians, some of them belonging to the working group delivering the intervention

c. **Setting**

• A low- income country, with limited but improving research capacities

d. **Mode of action**

• Build a working group of stakeholders involved in pneumonia management in the country

• Collect, appraise, analyze, synthesize data existing on pneumonia etiologies and antimicrobial profiles, in the literature and among the working group

• Collectively produce a corpus of evidence

• Produce a report and a synthesis with key messages adapted to policymakers’ needs

• Disseminate the report and synthesis

• Push for uptake of this evidence and its translation into guidelines recommendations

• Document the process

• Using:

– Direct interactions with the intervention recipients (meetings, correspondence),

– Written communications (full report and synthesis translated in local language, all available online)

– Data collection, analysis and synthesis, shared with the intervention’s recipients

e. **Duration**

• 8 months

f. **Adherence**

• Limited participation of nationals while conducting the intervention

2. **Assumed output**

a. Built up of shared knowledge on pneumonia based on local evidence

b. Uptake of the key messages about pneumonia etiologies and antimicrobial resistance and translation into the CPGs

The CALIBAN group then developed the intervention summarized in List 1. A systematic review and synthesis of literature on pneumonia etiologies and antimicrobial resistance levels in Cambodia and its neighboring countries was conducted [17]. The CALIBAN group then produced a comprehensive report including and an executive summary formatted according to the recommendations of the SUPPORT group to best accommodate policymakers’ needs [18]. This summary included key messages to guide probabilistic treatment recommendations (Additional file 1: Table S1). Documents were translated into the Khmer national language and were officially submitted by a national clinician from the CALIBAN group to the MoH in November 2012. These documents were also posted on a freely accessible website (http://www.webcitation.org/6Qo38T6HW).

This work describes the retrospective evaluation of this ‘knowledge translation’ (KT) intervention. KT is the process by which ‘stakeholders are aware of and use research evidence to inform their health and healthcare decision making’ [19]. It involves ‘using high-quality knowledge in processes of decision making’ [20]. Several authors have developed KT theories and frameworks [21-24] but the effectiveness and impact of KT interventions targeting policymakers are rarely reported [19].

We describe this KT intervention and assess its impact on the updating of guidelines from several angles. Our objective was to identify the barriers to KT encountered in this LIC setting, and to suggest strategies to facilitate KT in similar settings.

## Material and methods

### Data collection

We retrospectively searched for all relevant sources of data documenting the CALIBAN intervention and the pneumonia guidelines. We used contact with key informants, the IPC and CALIBAN archives, the National Institute of Public Health and MoH websites. Data collected included project protocols, reports, published literature, and meeting minutes from CALIBAN. It also included the successive drafts of guidelines, email correspondence between researchers, the CALIBAN network and the Task force, as well as notes taken during interviews with a key informant from the NGO who assisted the guidelines update. The review of these documents enabled us to describe and model the KT intervention.

### Analysis

#### Impact of the KT intervention on the updated guidelines

Firstly, we compared the CALIBAN key messages with the updated adult and pediatric guidelines released in 2013 to assess the extent to which the newly issued recommendations were in line with the key messages (Additional file 1: Table S1).

We also assessed how well the CALIBAN KT messages were integrated by examining the rigor and transparency of the guidelines updating process. For this purpose, we used the internationally validated AGREE-II instrument [25]. Each guideline was assessed by three appraisers as recommended by AGREE-II for an optimum reliability. Appraisers were clinicians and public health specialists who did not participate in the CALIBAN KT intervention. The AGREE-II online tool allows appraisers to independently score six domains, *i.e*., scope and purpose, stakeholder involvement, rigor of development, clarity of presentation, applicability, and editorial independence. The tool then sums up all the scores of appraisers and computes standardized domain scores (expressed on a scale of 0–100).

#### UML modeling

Secondly, we modeled and analyzed the CALIBAN KT process within the ‘guidelines updating’ system, using the Unified Modeling Language (UML) graphical tools. The purpose was to describe how this intervention worked and to provide a framework for assessing its effectiveness.

UML is an object-oriented modeling tool developed in 1997 for documenting, designing, and evaluating complex systems [26]. It is an effective medium of communication and development for both theorists and practitioners [27]. It has been used to model different aspects of healthcare systems [28,29] including the analysis of clinical trials [30].

**Figure 1:**
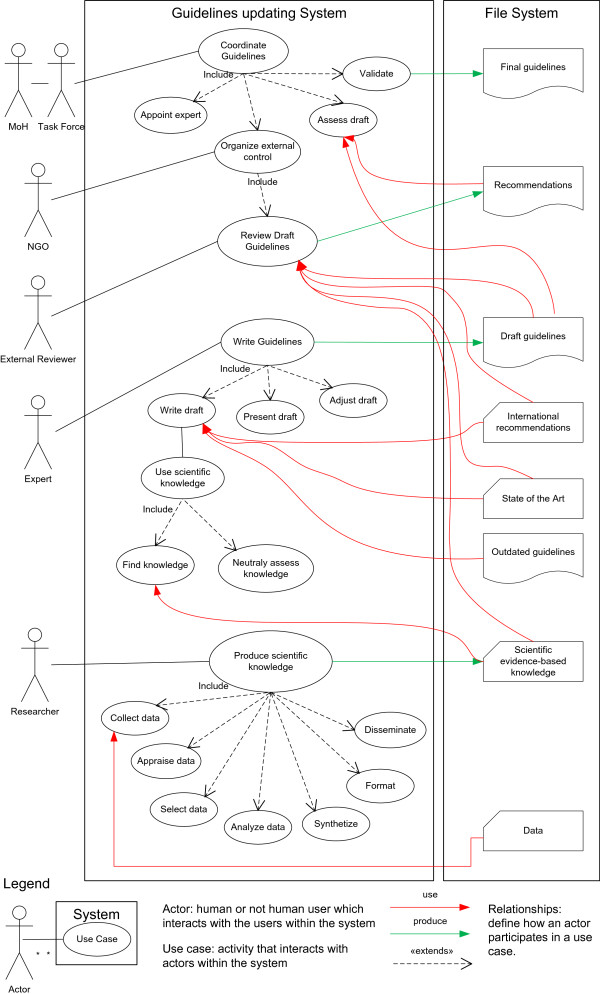
Use case—Knowledge translation during Clinical Practice Guidelines updating.

We initially developed a series of UML ‘use cases.’ A ‘use case’ represents a series of steps defining interactions between ‘actors’ and a ‘system,’ to successfully achieve a goal. The main ‘actors’ of the ‘guidelines updating’ system were identified and depicted as stylized human figures according to UML conventions (http://www.webcitation.org/6Qo2KJcEe) (Figure 1).

**Figure 2:**
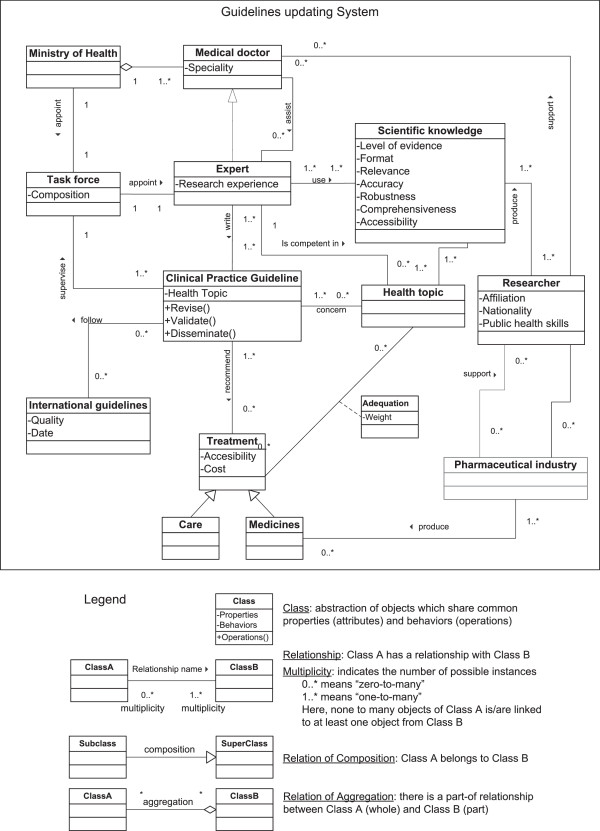
Class diagram—Knowledge translation and Clinical Practice Guidelines updating.

Then, we constructed a UML activity diagram displaying the sequence of activities conducted during the adult guideline’s updating (Additional files 2 and 3: Figures S1 and S2). We used this tool to frame and analyze the dynamics of activities, the interactions between actors and the documents’ exchanges during the adult guideline updating. We lacked the detailed information required to construct the same diagram for the pediatric guideline.Using both the UML ‘use cases’ and the UML ‘activity diagram’ we derived a structural class diagram describing the generic structure of the ‘guidelines updating’ system. The structural class enabled us to identify the system’s classes of objects involved in the system, their possible interfaces, attributes, collaborations, and relationships within and between the classes (Figure 2).

Modeling was performed in several successive steps using UML 2.2 Visio® Microsoft® software.

#### Identification of barriers and facilitators to KT

Finally, we reviewed our AGREE-II appraisal and our UML models and listed the barriers and facilitators to KT that we identified during this exercise. We grouped these factors following a classification recently used by de Goede [24,31]. Barriers at the process level are those encountered during the preparation phase and cover mutual expectations from researchers and policymakers, and those related to research findings communication (transfer). Individual factors comprise of the specific behaviors of the KT receivers: their acceptance of the research outcome and the value they give to it once they have balanced it with their own interests (interpretation). At the process level, we included in the existing classification some factors relating to the interactions between the stakeholders who produced the evidence on pneumonia (the CALIBAN group) and those who received and were supposed to use it (the MoH and Task forces).

### Ethical considerations

The CALIBAN project was based on anonymized acute lower respiratory infections data – including the IPC study [6,7] - gathered by research studies, all of which had obtained ethical clearance from the Cambodian National Ethics Committee for Human Research [17]. Our present article only examines the process through which the CALIBAN project was thought up and developed, and what obstacles were met. This paper is based on work which in no way involves interventions or research on human subjects or animals, nor do study results name any informants or stakeholders. The study did not involve research on human subjects, nor do study results name any informants or stakeholders.

## Results

### Guidelines content appraisal

The integration of the CALIBAN KT key messages varies across adult and pediatric guidelines (Additional file 1: Table S1).

The pediatric guidelines are well in line with those messages. They recommends antibiotics which CALIBAN found effective against the most prevalent bacterial pathogens responsible for pneumonia in Cambodia (penicillin A for non-severe cases; penicillin A and gentamicin as first-line treatment for severe cases, with a switch to ceftriaxone in case of no improvement or to cloxacillin if staphylococcal pneumonia is suspected). It also refers to additional guidelines to treat pneumonia due to the less frequent pathogens cited by CALIBAN and which require specific treatments.

Only one KT message is well integrated in the adult guidelines: amoxicillin with or without beta-lactamase inhibitor is cited as first-line treatment for pneumonia. The guideline cites atypical pathogens as possible pneumonia etiologies although the CALIBAN group could not firmly document their prevalence in the country. The second-line option given for non-severe pneumonia are macrolides which are not effective against the most prevalent pathogens in adult pneumonia in Cambodia. Macrolides are also inappropriately recommended as single-drug for patients hospitalized in intensive care with very severe pneumonia. Moreover, the guidelines recommend fluoroquinolones for first-line treatment for severe pneumonia in hospitalized patients and for second-line or alternative treatment for uncomplicated cases. These fluoroquinolones are expensive, have side effects, and should be preserved to avoid resistance and remain a powerful tool against tuberculosis, which is highly prevalent in Cambodia. Finally these guidelines do not mention the possible use of Amikacin in case of severe pneumonia due to Gram-negative bacteria, although this was among the CALIBAN key messages.

### AGREE-II appraisal

The AGREE-II assessment showed that both guidelines did not meet some development and editorial independence standards of rigor (Table 1). Rigor relates to the source of evidence on which recommendations are based. It scored 16% and 10% for adult and pediatric guidelines respectively. The appraisers noted the absence of reference to local and regional studies on pneumonia. The CALIBAN summary was annexed to both guidelines, only at the insistence of the supporting NGO and high-ranking MoH officials, and no reference was made to it within the text. Seven out of the nine references cited in the adult guidelines were Western medical textbooks and data from Western countries. The pediatric guidelines cited three WHO documents, one Western guideline, and one scientific article on pneumonia (not open access and with the study area not mentioned in the abstract).

**Table 1:** AGREE-II scores for the adult and the pediatric pneumonia guidelines (standardized domain scores across appraisers), Cambodia 2013

	**Clinical practice guidelines**		
**Domain of AGREE II**	Adult	Pediatric	Mean
Presentation of scope and purpose	49%	65%	57%
Stakeholder involvement	37%	31%	34%
Rigor of development	16%	10%	13%
Clarity of presentation	54%	69%	62%
Applicability	18%	38%	28%
Editorial independence	12%	11%	12%
**Overall assessment**	**37%**	**44%**	**41%**

The AGREE-II criteria on editorial independence scored 12% and 11% for adult and pediatric guidelines, respectively. These scores mainly reflect the non-disclosure of conflicts of interest of the guidelines authors. Potential conflicts of interest such as those from drug companies could not be assessed because they were not disclosed.

### Modeling analysis of the guideline updating process

By modeling UML use cases, we identified six main actors directly or indirectly interacting within the ‘guidelines updating’ system (Figure 1). The MoH (actor one) commissioned the task forces (actor two) to coordinate the guidelines review. The NGO (actor three) provided logistical support to the task forces and organized the external review. The experts (actor four) were the clinician task forces’ members who led the work and wrote the guideline drafts. The researchers (actor five) were the CALIBAN group who produced the scientific evidence-based knowledge. The external reviewers (actor six) reviewed the guidelines’ drafts.

The activity diagram shows the activities of four actors directly involved in the updating of the adult pneumonia guidelines (Additional file 3: Figure S2). It illustrates how all four actors interacted several times. It also highlights that actors missed several opportunities to base this adult guideline on evidence (n = 6 red dots in the figure). The time frames and constraints of the various stakeholders involved were not the same. Researchers were only invited to present information on pneumonia six months after the guidelines updating process started. The experts urged researchers to communicate their results to finalize their task and meet the deadlines, while the researchers, bound to scientific methodology and ethics could not release preliminary and incomplete findings while the review was under way. Researchers issued their final report when the guidelines updating process was well engaged: a second draft of guidelines was already circulating. This draft was validated four months later without any change. Conversely to what happened with the adult guidelines, the main expert in charge of updating the pediatric guideline had a long history of collaboration with pneumonia researchers and endorsed the modifications suggested by the external reviewers.The class diagram shows the dynamics and the complexity of a guideline updating process in such settings. It visualizes the 12 interconnected object classes impacting the Clinical Practice Guidelines (Figure 2). Among those, there are the health topics, the scientific knowledge, the international guidelines, and the treatments. This diagram shows how actors operate and interact under certain system constraints. It brought out the potential role of pharmaceutical industries that produce the medicines. These industries might influence researchers’ and experts’ judgment by supporting them. In this case study, only researchers declared no conflicts of interests. Experts did not disclose any.

### Facilitating and impeding factors to KT

We identified 17 facilitating factors and 18 potential barriers to KT (Table 2). Most facilitating factors and barriers were identified at the KT process level (point 1, Table 2, n = 13/17 facilitators, and n = 14/18 barriers), while fewer were found at the Individual level (point 2, Table 2, n = 4/17 facilitators and n = 3/18 barriers).

**Table 2:** Facilitating factors and barriers to KT during the pneumonia guidelines updating, Cambodia 2013

**Facilitating factors**	**Barriers**
**1**- **Process level**	
**1.1 Expectation domain**	
**1.1.1 Research production**	
1. KT intervention provided relevant, accurate, robust, comprehensive and accessible information to Policymakers	1. The Task force had not planned to request local evidence from locally-based researchers
**1.1.2 Timing**	
2. Being members of the KT intervention group, the Experts had a privileged access to research findings before the final results were available	2. Limited availability of the Task force -busy with the updating of about 200 guidelines chapters at the same time
	3. MoH did not initially inform the Researchers of the guidelines review process
	4. The evidence readily available at the initiation of the guidelines updating process was not complete enough to be used by Policymakers
	5. Researchers started working on building evidence seven months after the process was initiated by the MoH
	6. Policymakers expected the Researchers to provide some evidence quicker.
**1.1.3 Policy process**	
3. WHO facilitated the contact between Researchers and Policymakers: WHO informed the Researchers that the guidelines were updated	7. Patients’ representatives were not associated to the process. They could not relay the need to base the Guidelines updating on local evidence
4. Policymakers received support from an international NGO for some organizational aspects of the process (organization of the Task force meetings, of the External Review Committee…)	8. Limited availability of clinicians with expertise. (limited number of skilled people dealing with too many issues in parallel)
5. The NGO assisting the Policymakers successfully relayed Researchers’ demand to annex the KT messages to the CPGs	9. The Task force left the Experts deciding to accept or refuse the External Review Committee’s suggestions for improving the final drafts of guidelines. The Expert in charge of the adult pneumonia guidelines did not accept changes suggested by the External Committee
6. The Expert who led the work on the pediatric guideline agreed to take into account the recommendations issued by the External Review Committee	
**1.2 Knowledge Translation domain**	
7. Research synthesis included key messages	10. No communication was released to the media by the Researchers. Therefore the process did not receive any media support
8. Research synthesis was written in plain and easy English and translated into local language	11. Research synthesis and report did not present any logo at their front page, except the logo of the KT group. This may have limited the identification of authors and their perceived credibility (but facilitated the easy appropriation by all co authors)
9. Research synthesis was short and compliant with the SUPPORT recommendations	
10. Research synthesis was widely made permanently available online	
**1.3 Interactions between policymakers and researchers**	
**1.3.1 Initiated by Policymakers during Guidelines Updating process**	
11. Policymakers invited the Researchers to participate in one of their meetings	12. Participation of Researchers in the Task force was limited to one meeting
12. A clinician who was familiar with Research was appointed by the Task force to update the pediatric pneumonia guidelines. This clinician had a long lasting history of collaboration with pneumonia researchers	13. The Expert appointed for the adult pneumonia guideline review had no or limited previous interactions with Researchers
**1.3.2 Initiated by Researchers during the KT intervention**	
13. Researchers invited national clinicians and Experts appointed by the Task force to participate in the KT intervention	14. Meetings organized during the KT intervention were conducted in English which is not the working language of most clinicians in Cambodia
	15. Researchers’ attempts to alert on inappropriate recommendations published in the adult pneumonia guidelines remained unanswered
**2**- **Individual level**	
**2.1 Acceptance domain**	
**2.1.1 Perceived robustness of evidence**	
14. Researchers clearly stated the limitations of their Evidence review in their synthesis	16. Researchers do not know how the robustness of their findings was perceived by Policymakers
**2.1.2 Perceived credibility of source**	
15. Data contributing to the KT intervention were provided bystakeholders known by the policymakers	
**2.1.3** ‘**Fit**’ **with personal knowledge**, **values or belief systems**, **preference and traditions**	
16. Researchers analyzed data in the light of current challenges for the national health system (prevention of development of antibiotic resistance, cost effectiveness)	17. There is not much mutual knowledge on values, belief systems, preference and traditions between Researchers and Policymakers
**2.2 Interpretation domain**	
**Connection with own personal or institutional interests**	
17. Researchers declared no conflicts of interest in their evidence review	18. Policymakers did not disclose potential conflicts of interest in the guidelines

Most barriers to KT encountered were related to the lack of synchronization between knowledge production and policy making (Point 1.1.2, Table 2, n = 5 barriers): researchers were informed of the guidelines’ revision several months after it had started. They had no formal mandate from the MoH. Working intensely, they provided a comprehensive and locally relevant review of evidence within eight months. However, this pace did not match the policymaker’s time constraints. We also identified barriers at the policy-making process (Point 1.1.3, Table 2, n = 3 barriers). Those factors are intrinsic to the current situation in Cambodia, such as the limited availability of clinicians with expertise, or are more individual-dependent (*e.g*., barrier n.9, Table 2). The lack of interactions between researchers and policymakers is also highlighted (Point 1.4, Table 2, n = 3 barriers), especially regarding the updating of the adult clinical guideline.

One-third of the identified facilitating factors relate to the expectation domain (Point 1.1, Table 2, n = 6 factors). In particular, the policy process received help from several external stakeholders: the WHO, NGO and the External review committee (Point 1.1.3, Table 2, n = 4 facilitators). Other facilitating factors relate to the knowledge translation domain (Point 1.2, Table 2, n = 4 facilitators).

The difference between the process of updating the pediatric and the adult guidelines bears on only two points: the pediatric expert had a long term collaborative experience of with researchers in pneumonia research projects, while the expert working on the adult guideline had very little or none; the pediatric expert agreed to take into account the suggestions made by the external review committee while the adult expert argued that those suggestions came too late in the process.

## Discussion

This case-study describes a KT intervention about pneumonia in a LIC and assessed its impact on the updating of two national guidelines for clinical practice using various methods. Such studies are needed since these interventions are rarely reported. Moreover, there is no consensual guidance to date on how to update national guidelines in such countries [13,19]. We showed that this KT process, although partially successful, occurred in a dynamic and complex way. Comparing the KT process during the updating of both pediatric and adult pneumonia guidelines allowed us to identify possible facilitating and impeding factors to KT in similar situations.

The pneumonia knowledge produced by the CALIBAN group was successfully translated into the pediatric guidelines. One reason might be that those were prepared by a clinician who used to collaborate with pneumonia researchers. Findings are consistent with what was described elsewhere: when policymakers have a long and strong experience of collaboration with researchers, knowledge utilization is most likely to occur [32,33]. This principle drives the KT ‘interaction model’ considered as an effective model for optimizing research use [31]. When policymakers are aware of research constraints, limitations, and strengths, they likely incorporate these into policies [33]. Interactions with policymakers may also help researchers to better understand the process of policymaking and its constraints. This ‘interaction model’ takes into account the complexity of the health systems in their context and the interactive and incremental nature of policy development.

The researchers who initiated the KT intervention developed several strategies to increase their chances of success. First, they invited the clinicians in charge of writing the guidelines to chair and participate in developing the KT intervention. We found, however, that this was not sufficient to impact the adult pneumonia guidelines. Similarly, a study in Canada showed that when policymakers are only involved in the synthesis of research findings, they better understand these findings but do not necessarily use them [33].

Second, CALIBAN researchers worked on the quality of the evidence they intended to transfer. They naively expected that providing only relevant, comprehensive, and robust evidence to the right persons would be sufficient to influence the policymaking. This assumption drives the ‘KT push-model’ [34] that was found to be inefficient: knowledge utilization does not only depend on supply of research findings [21,22,24]. Indeed, we showed that the scientific knowledge supplied by the researchers did not spontaneously and simply climb what is called the ‘ladder of knowledge utilization’ [34]. CALIBAN findings were transmitted to relevant stakeholders (first stage of the ladder). Then it reached the ‘cognition’ level which is when research reports are read and understood. But those reports only reached the ‘reference’ echelon (reports are cited) after a strong encouragement from various stakeholders. Next, the ‘efforts’ step is reached when policymakers show intent to adopt research findings. In this case study, we showed that the ‘influence’ level (when results have influenced choices and decisions) was actually reached only in the case of the pediatric guideline. ‘Application,’ the ultimate step, is reached when the evidence gives rise to application and extension by program managers. This ultimate step may never be reached.

The last strategy developed by the researchers was to format their messages to meet the expected needs of the health policymakers following international and recognized recommendations [18]. Indeed, this strategy is reported as a major facilitating factor by policymakers by Innvaer’s systematic review of 24 studies [32]. Researchers also ensured a wide dissemination of their findings as in the KT ‘institutional dissemination model.’ However, as shown elsewhere, it probably increased the chances of the CALIBAN messages being integrated into the guidelines, but was not sufficient [34].

Most barriers to KT we identified were related to the poor synchronization between knowledge production and policy making, and to the lack of mutual understanding between researchers and policymakers.

The timing of activities was a major issue in this KT intervention. The lack of timeliness on the production of research evidence is frequently perceived as an important barrier for the effective use of research data, even in developed countries [32]. Developing long-term collaborations between researchers and policymakers would probably help overcoming this barrier.

Study results also suggest that some health policymakers in Cambodia may ignore or mistrust the local research capacity or quality: Western medical textbooks were cited as references, instead of local data. This may be due to the lack of appreciation of the value of locally generated evidence, emerging from poor interactions between researchers and policymakers. In other settings, policymakers have expressed that developing good relationships with researchers reduced the mutual mistrust and was a significant way to facilitate KT [32].

Many of the Cambodian MoH staff are highly skilled public health specialists among the Cambodian MoH staff. Involving these Cambodian public health specialists may have prevented the recommendations of non-adapted and expensive treatments that can jeopardize public health, as observed in the adult pneumonia guidelines [35].

It was difficult to assess the relative importance of the barriers to KT and what determines them, mostly because of the limited transparency in the updating of the guidelines. The AGREE-II appraisal highlighted the possible lack of rigor of development and of ‘editorial independence.’ Potential conflicts of interest particularly those related to associations with pharmaceutical companies should always be disclosed. Those companies might be tempted to use guidelines to promote the use of their products and therefore to influence expert judgment [36]. Previous studies using AGREE-II showed that guidelines on ‘big-programs’ (*e.g*., HIV, malaria) scored better, demonstrating the positive effect of funding and international attention [15,37].

The role of external stakeholders such as the WHO and, the NGO who assisted the task forces and the external review committee was noted as a key facilitating factor of KT in this study.

The WHO and the NGO played a crucial communication role, first of all by informally informing the researchers about the MoH agenda to update guidelines and later by liaising between researchers and policymakers. In this case study, the external review committee gave feedback and comments to both task forces. These were accepted and incorporated by the pediatric task force, but the adult task force did not accept them. Recent recommendations on development of guidelines indicate that a review of the a final drafts must be conducted by clinicians and methodological experts not involved in preparing the guidelines [13].

### Lessons learned

From the discussion above, we draw a few lessons and tentatively make recommendations to researchers and policymakers seeking to facilitate the KT from research to health policies and guidelines.

### To researchers

1. Consult policymakers about their public health priorities to define research questions and do not only base research questions on literature reviews or the advancement of science.

2. Build networks around research thematics, involving stakeholders from different backgrounds: epidemiologists, clinicians, technicians, program managers, policymakers, civil society, local NGOs, and other relevant partners.

3. Work on the timeliness and the relevance of evidence shared with policymakers in addition to accuracy and robustness.

4. Besides scientific articles and progress/finding reports, prepare findings synthesis, following the SUPPORT format http://www.webcitation.org/6Qo2OBAXg. Structured summaries must include key messages, sources and methodology, scope and limits of the results and conclusions. Summaries should be presented in person to key stakeholders, with time for discussion, rather than simply handed out.

### To policymakers

1. Plan the work on the national clinical guidelines ahead of time and formally request data from researchers. Consider the time it takes to obtain the most robust, comprehensive, and therefore relevant data on which the guidelines must rely.

2. Publicize the process for developing/updating of guidelines. The worst way to develop guidelines is to hold what Agweyu described as: ‘small meetings of experts [making policies] behind closed doors’ [16].

3. Seek methodological support. Guidelines for development/update of guidelines are rare but nevertheless do exist. In 2007, the ADAPTE collaboration developed a systematic approach, a manual and tools to facilitate the adaptation of existing guidelines to different contexts (http://www.webcitation.org/6Qo0xEWDn). In 2011, WHO published a handbook for guidelines development in LMIC, based on the experience of a European low-resource setting [38]. Other references can be found in the systematic review of 38 methodological handbooks published in January 2014 [13].

4. Involve external experts and officially mandate an External Review Committee.

5. Define a roadmap for the Experts in charge of drafting the guidelines, describing the way to interact with other stakeholders involved in the process (in particular the external review committee).

### To both policymakers and researchers

1. Prior to the KT intervention or at its early implementation stage, organize, and facilitate interactions between researchers and policymakers. This potentially makes policymaking and research more compatible.

2. ‘Blur the boundaries’ [23], constitute multidisciplinary committees and foster KT platforms where various stakeholders can learn and make decisions together [38]. Make policymakers and the researchers aware of each other’s agendas, constraints, and objectives. Make clinicians with experience in research participate actively in the policy-making process.

3. Identify and involve effective KT coordinators/facilitators—sometimes called ‘knowledge brokers’ [39]. Their tasks could be to organize meetings, liaise with involved stakeholders, retrieve and format the evidence needed to support the guidelines, prepare recommendation matrices, facilitate consensus, prepare guideline drafts, and coordinate internal and external reviews [40].

4. Document and report additional case reports to further support the development of guidelines for developing/updating guidelines.

Finally there are also some institutional lessons that could be derived from this case report:

1. Declaring conflicts of interest should be mandatory for researchers, tasks forces, and expert committees involved in guideline development, especially with regard to guidelines that make recommendations on medication alternatives.

2. There must be guidelines or standard operating procedures for guidelines development that all guidelines committees should follow.

3. The use of available evidence as well as the grading of evidence should be part of the above guidelines.

4. Task forces should be provided with tools to grade locally acquired evidence. Otherwise they may overstate the contribution of western textbooks.

### Limitations

The main limitation of this analysis is that it was conducted from the perspective of those who designed and implemented the KT intervention since it was the only source available. We used the quality and content appraisals of the updated pneumonia guidelines as a proxy for KT impact. Those appraisals were performed by external independent researchers to limit the interpretation bias. The modeling analysis was conducted by five professionals from various backgrounds to also limit potential bias of analysis. Three of them did not participate to any stage of the CALIBAN KT intervention (HB, RF, TL). Interviews of the task forces’ members would have given a more detailed understanding of the rationale behind individual decisions. Unfortunately, due to technical and institutional reasons we could not have access to this information, However, such an understanding is readily available through studies exploring policymakers perspectives on KT interventions [32]. An additional element of complexity is that some persons with a key role in the guidelines updating process were also knowledge dispensers.

## Conclusions

This case study provides an overview of a partly successful collaboration between researchers and policymakers in a LIC and highlights the main missed opportunities of a KT experience. Longterm and close interaction between researchers and policymakers was the main facilitating factor of KT. Other efforts made by the researchers such as building accurate robust local evidence, formatting it to the needs of policymakers, inviting policymakers to participate in the KT intervention were not sufficient. Stakeholders—policymakers and researchers—planning to update clinical guidelines in LIC should receive methodological support to overcome the expected barriers.

## Abbreviations

WHO: World Health Organization; MoH: Ministry of Health; NGO: Non-governmental organization; CPG: Clinical practice guidelines; KT: Knowledge translation.

## Competing interests

All authors declare that they have no competing interests.

## Authors’ contributions

SG led the data collection and analysis and wrote the manuscript. RF, HB, and AT iteratively commented the manuscript drafts and oversaw its development. HB, TL, JvG, RF, and AT were involved in the data analysis. TL oversaw the UML data modeling. AT and SG initiated and led the CALIBAN intervention. JvG was a CALIBAN member. All authors critically read the manuscript and have given final approval. The views expressed in this article are those of the authors and not necessarily those of their institutions. All authors declared no conflicts of interest.

## Supplementary Material

Additional file 1: Table S1 CALIBAN key messages *vs* 2013 Pneumonia guidelines. Click here for file

Additional file 2: Figure S1 Legend of the Activity diagram. Click here for file

Additional file 3: Figure S2 Unified Modeling Language Activity diagram - Knowledge translation and Clinical Practice Guidelines updating. Click here for file
