# Supplementary figures and images for: Knowledge translation: a case study on pneumonia research and clinical guidelines in a low- income country
Source: Implement Sci. 2014 Jun 26;9:82. doi: 10.1186/1748-5908-9-82 (PMC4094455; doi:10.1186/1748-5908-9-82)

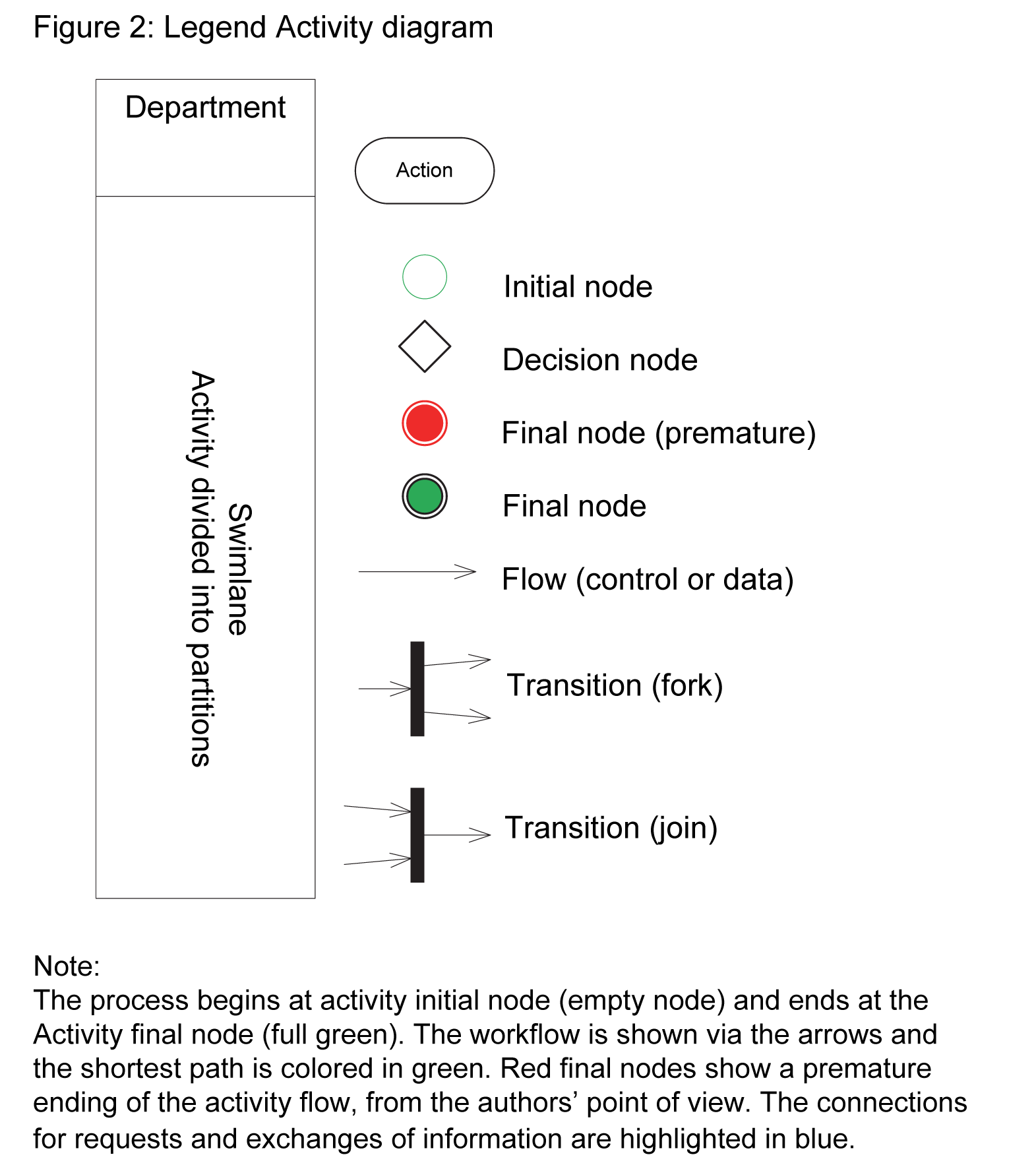

Supplement: Additional file 2: Figure S1 — Legend of the Activity diagram. [file 1748-5908-9-82-S2.tiff]

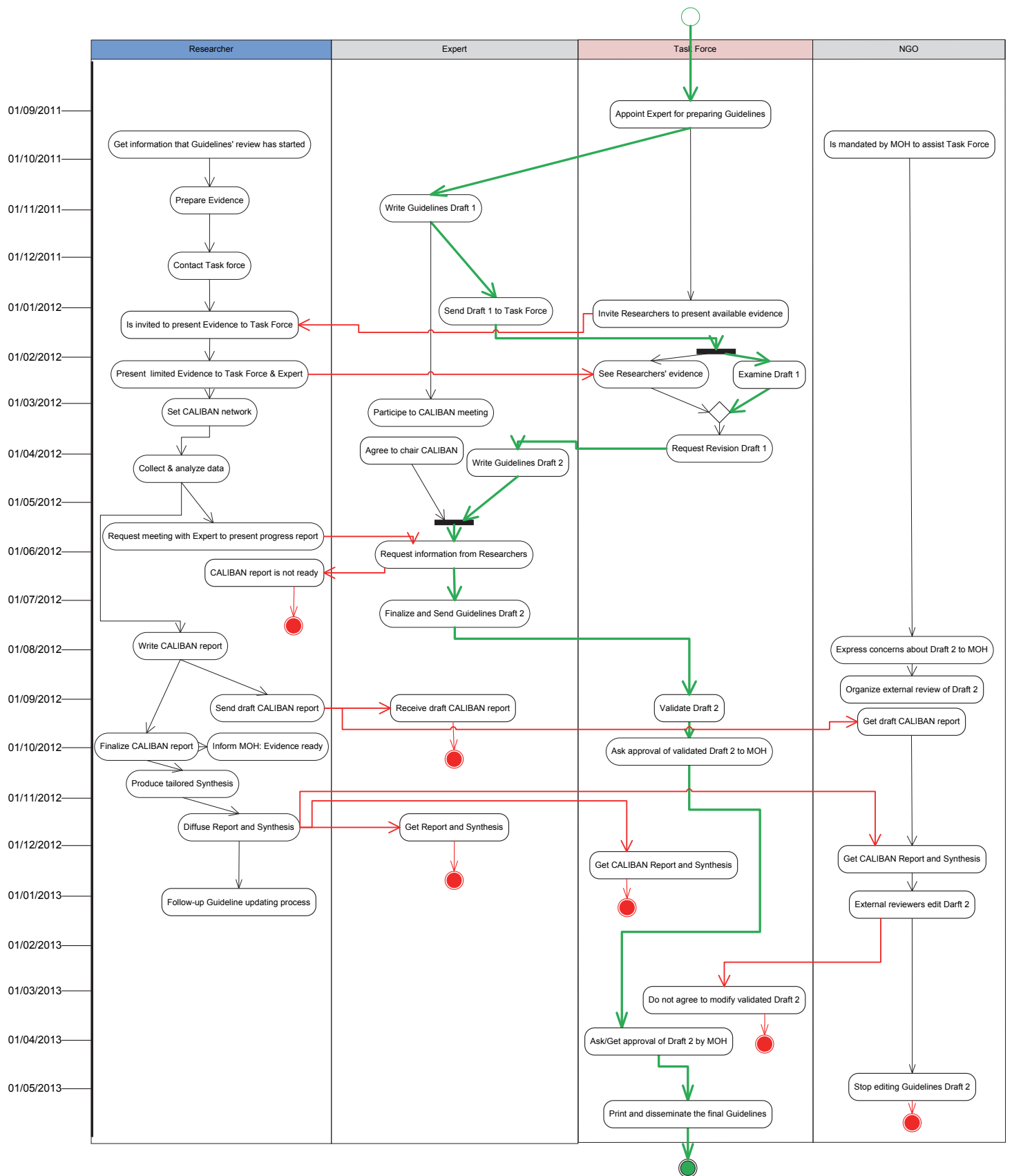

Supplement: Additional file 3: Figure S2 — Unified Modeling Language Activity diagram - Knowledge translation and Clinical Practice Guidelines updating. [file 1748-5908-9-82-S3.pdf]
